# Endogenous Sulfur Dioxide: A New Member of Gasotransmitter Family in the Cardiovascular System

**DOI:** 10.1155/2016/8961951

**Published:** 2015-12-29

**Authors:** Yaqian Huang, Chaoshu Tang, Junbao Du, Hongfang Jin

**Affiliations:** ^1^Department of Pediatrics, Peking University First Hospital, Beijing 100034, China; ^2^Department of Physiology and Pathophysiology, Peking University Health Science Centre, Beijing 100191, China; ^3^Key Laboratory of Molecular Cardiology, Ministry of Education, Beijing 100191, China

## Abstract

Sulfur dioxide (SO_2_) was previously regarded as a toxic gas in atmospheric pollutants. But it has been found to be endogenously generated from metabolism of sulfur-containing amino acids in mammals through transamination by aspartate aminotransferase (AAT). SO_2_ could be produced in cardiovascular tissues catalyzed by its synthase AAT. In recent years, studies revealed that SO_2_ had physiological effects on the cardiovascular system, including vasorelaxation and cardiac function regulation. In addition, the pathophysiological effects of SO_2_ were also determined. For example, SO_2_ ameliorated systemic hypertension and pulmonary hypertension, prevented the development of atherosclerosis, and protected against myocardial ischemia-reperfusion (I/R) injury and isoproterenol-induced myocardial injury. These findings suggested that endogenous SO_2_ was a novel gasotransmitter in the cardiovascular system and provided a new therapy target for cardiovascular diseases.

## 1. Introduction

Sulfur dioxide (SO_2_) was regarded as a toxic gas and environmental pollutant. It is colorless, transparent, odorous, and water-soluble. The harmful effects of SO_2_ on human, animals, and plants have been extensively investigated [1, 2]. However, SO_2_ can be endogenously generated from metabolism of the sulfur-containing amino acid L-cysteine in mammals [3]. It has features of low molecular weight, continuous production, and fast diffusion and plays extensive biological action independent of membrane receptors [4, 5]. In neutral fluid or mammal plasma, SO_2_ is broken down to its derivatives, bisulfite and sulfite (NaHSO_3_/Na_2_SO_3_, 1 : 3 M/M), maintaining organism homeostasis [6]. The sulfite is the physiological form of SO_2_ *in vivo* [7, 8]. The reference range for total serum sulfite in healthy human beings was 0–9.85 *μ*mol/L detected by high-performance liquid chromatography with fluorescence detection [9]. Serum sulfite was obviously increased in patients suffering from acute pneumonia and chronic renal failure, as well as pediatric acute lymphoblastic leukemia with bacterial inflammation [10–12]. Of note, Balazy et al. found that SO_2_ could be produced in the porcine coronary arterial rings after incubation with calcium ionophore by gas chromatography-mass spectrometry [13]. Du et al. firstly found that endogenous SO_2_/aspartate aminotransferase (AAT) pathway existed in the cardiovascular system [14]. SO_2_ not only has important physiological effects on vascular tone and cardiac function but also exerts pathophysiological effects in the cardiovascular system, including regulation of hypertension, pulmonary hypertension, atherosclerosis, and cardiac ischemia-reperfusion (I/R) injury [15–18]. The abovementioned evidence suggests that the endogenous SO_2_ may be a novel gasotransmitter in mammals, similar to nitric oxide (NO), carbon monoxide (CO), and hydrogen sulfide (H_2_S). The physiological significance of SO_2_, particularly its regulatory role in the cardiovascular system, has attracted a great deal of interest in the field [19–21].

Therefore, the objective of this review was to elaborate on the generation and metabolism of endogenous SO_2_ and give a summary of the physiological and pathophysiological effects of SO_2_ on the cardiovascular system.

## 2. Generation and Distribution of Endogenous SO_**2**_ in the Cardiovascular System

**Figure 1:**
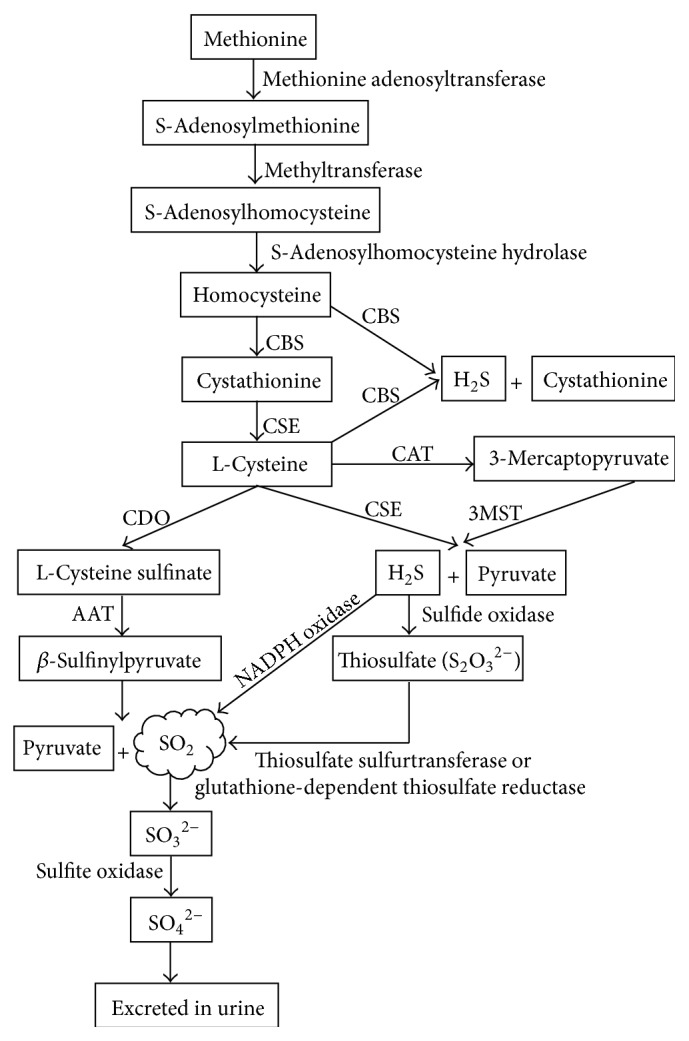
Illustration of production and metabolism of endogenous SO_2_ in mammals. SO_2_: sulfur dioxide; H_2_S: hydrogen sulfide; CBS: cystathionine *β*-synthase; CSE: cystathionine *γ*-lyase; CDO: cysteine dioxygenase; AAT: aspartate aminotransferase; CAT: 2-cysteine aminotransferase; 3MST: 3-mercaptopyruvate sulfurtransferase.

SO_2_ can be generated from the metabolism of L-cysteine which is converted from methionine via the transmethylation-transsulfuration pathway (Figure 1) [3, 22]. Firstly, L-cysteine is oxidized to form L-cysteine sulfinate by cysteine dioxygenase (CDO), and then the latter is transaminated to form *β*-sulfinylpyruvate by AAT. The *β*-sulfinylpyruvate spontaneously decomposes to pyruvate and SO_2_ (Figure 1) [3]. Additionally, H_2_S which shares the same substrate L-cysteine with SO_2_ can be transferred to SO_2_ *in vivo* through other pathways. Mitsuhashi et al. reported that H_2_S could be converted to sulfite or SO_2_ by NADPH oxidase in activated neutrophils [23]. Besides, H_2_S can be first oxidized to thiosulfate by sulfide oxidase and then converted to SO_2_ catalyzed by thiosulfate sulfurtransferase or glutathione-dependent thiosulfate reductase (Figure 1) [6, 24, 25]. SO_2_ can exist in the gaseous form or be hydrated to sulfite, which is subsequently oxidized to sulfate by sulfite oxidase, and then the sulfate is excreted into the urine by the kidney (Figure 1) [3, 22].

Du et al. first measured endogenous SO_2_/AAT pathway in the cardiovascular system of Wistar rats and found that SO_2_ concentration in rat plasma was 15.54 ± 1.68 *μ*mol/L [14]. Li and Meng reported a similar sulfite level of 12.59 ± 9.03 *μ*mol/L in rat plasma [26]. The content of SO_2_ in aortic tissue was highest, up to 5.55 ± 0.35 *μ*mol/g protein, followed by pulmonary arteries (3.27 ± 0.21 *μ*mol/g protein), mesenteric arteries (2.67 ± 0.17 *μ*mol/g protein), tail arteries (2.50 ± 0.20 *μ*mol/g protein), and renal arteries (2.23 ± 0.19 *μ*mol/g protein), respectively [14]. Moreover, plasma AAT activity was 87 ± 18 U/L. Unlike SO_2_ content, the activity of AAT in the renal arteries was higher than that in other vascular tissues mentioned above [14]. Furthermore, AAT mRNA expression was rich in endothelial cells and in vascular smooth muscle cell (VSMC) beneath the endothelial layer [14].

## 3. Physiological Effects of SO_**2**_ on the Cardiovascular System

### 3.1. Vasorelaxant Effect of SO_2_

SO_2_ derivatives (mixture of sodium bisulfite and sodium sulfite, 1 : 3 M/M in neutral solution) could induce a concentration-dependent relaxation of isolated rat aortic rings, whereas L-aspartate-*β*-hydroxamate (HDX), an inhibitor of SO_2_ synthase AAT, caused greater vasoconstriction than that of the control group [14]. And the vasorelaxing effects of SO_2_ gas and SO_2_ gas solution were similar [27, 28]. Therefore, SO_2_ might act as a vasoactive molecule. It had a vital vasodilating function required for maintaining normal vascular tone.

The mechanisms of this physiological vasorelaxation by SO_2_ were complex. Nicardipine eliminated the vasorelaxing effect induced by SO_2_ derivatives, indicating that the L-type calcium (L-Ca^2+^) channel participated in the role of SO_2_ [14]. Additionally, at low concentration (<450 *μ*mol/L), the vasorelaxing effect of SO_2_ was related to the big-conductance Ca^2+^-activated K^+^ (BK_Ca_) channel, while at a high concentration (>500 *μ*mol/L) the vasorelaxation induced by SO_2_ was associated to adenosine triphosphate-sensitive potassium (K_ATP_) channel activation and the L-Ca^2+^ channel [29]. Mechanistically, SO_2_ and its derivatives induced the K_ATP_ and BK_Ca_ channels activation through increasing the expressions of Kir6.1, Kir6.2, SUR2B, and BK_Ca_ channel subunits *α* and *β*1 in rat aortic rings, while SO_2_ and its derivatives inhibited the L-type calcium channel through decreasing the expressions of Cav1.2 and Cav1.3 [30]. Besides, SO_2_ derivatives increased levels of 3′-5′-cyclic adenosine monophosphate (cAMP), prostacyclin (PGI_2_), adenylyl cyclase (AC) activity, and protein kinase A (PKA) activity in rat aortic rings, indicating that the relaxing effect of SO_2_ was related to the PGI_2_-AC-cAMP-PKA signal pathway [31, 32]. Moreover, the endothelial nitric oxide synthase- (eNOS-) nitric oxide- (NO-) 3′-5′-cyclic guanosine monophosphate (cGMP) pathway and BK_Ca_ channel partially mediated the vasorelaxing effect of SO_2_ and sodium bisulfite in an endothelium-dependent manner at low concentration (<450 *μ*M), while at high concentration (≥1000 *μ*M) the vasorelaxation induced by SO_2_ was endothelium independent and relied on the K_ATP_ and L-Ca^2+^ channels [26, 33, 34]. Hence, ion channels, such as L-Ca^2+^, K_ATP_, and BK_Ca_ channels, as well as cGMP and cAMP pathways play important roles in the effects of SO_2_ on vasodilation.

### 3.2. Negative Inotropic Effect of SO_2_

In isolated perfused rat heart, gaseous SO_2_ and its derivatives (NaHSO_3_/Na_2_SO_3_, 1 : 3 M/M, 0–2000 *μ*mol/L) elicited a dose-dependent negative inotropic effect, which affected the heart rate, left ventricular developed pressure (LVDP), and the first derivatives of LVDP (±LV *dp*/*dt*_max_) [35, 36]. And the gaseous SO_2_ induced a server negative effect compared to SO_2_ derivatives. The mechanisms for this inotropic effect are different between high concentration and low concentrations of SO_2_. At low concentrations, SO_2_ produced negative inotropic effects through upregulating the activities of protein kinase C (PKC), cyclooxygenase, and cGMP, while, at high concentrations, the inotropic effects induced by SO_2_ were associated with the activation of K_ATP_ channel by increasing the expressions of Kir6.2 and SUR2A and the inhibition of calcium influx via the L-type calcium channel by decreasing the expressions of Cav1.2 and Cav1.3 in rat hearts [36, 37]. Moreover, SO_2_ could depress L-type calcium channel current in isolated rat cardiomyocytes [38]. These data indicated that SO_2_ had a negative inotropic effect on myocardial contractility and hemodynamic parameters, which might help to explain some cardiovascular effects induced by SO_2_.

## 4. Pathophysiological Effects of SO_**2**_ on the Cardiovascular System

### 4.1. SO_2_ and Hypertension

Hypertension is a major risk factor for many cardiovascular disorders. However, the pathogenesis of hypertension has not been fully elucidated. Exposure to SO_2_ (50 ppm, 6 hr/d, 5 d/wk for 31 weeks) was reported to cause a slight but consistent decrease in blood pressure in susceptible to salt-induced hypertension rats [39], implying that SO_2_ might regulate blood pressure. Moreover, spontaneously hypertensive rats (SHRs) exhibited a significant decrease in the plasma SO_2_ content and AAT activity in both serum and aorta [15]. And SO_2_ derivatives administration markedly inhibited the upregulated tail artery pressure of SHRs [15, 40], which suggested that SO_2_ played a role in the progress of hypertension. Arterial remodeling predominates in severe hypertension [41]. As well, SO_2_ alleviated the pressure to media, decreased the ratio of media to lumen radius, and reduced the proliferative index of smooth muscle cells in the thoracic aorta of SHRs compared to those of sterile water-treated rats [15]. These findings further verified that the inhibited endogenous SO_2_/AAT pathway might participate in the development of hypertension. Vasorelaxation dysfunction is the main component of the pathogenesis of hypertension. SO_2_ could increase vasorelaxation in SHR arteries by enhancing the vasodilating response to NO in isolated aortic rings and promoting NO production of aortic tissues [40]. The interaction between SO_2_ and NO is involved in the mechanisms by which SO_2_ regulates hypertension.

The abnormally increased proliferation of VSMCs induces vascular remodeling and accelerates the development of hypertension [42]. Both exogenous SO_2_ derivatives and endogenous-derived SO_2_ by AAT overexpression significantly inhibited serum-stimulated VSMC proliferation through preventing cell cycle progression from G1 to S phase and inhibiting DNA synthesis [43]. Further study demonstrated that SO_2_ elevated cellular cyclic adenosine monophosphate (cAMP) production to activate the PKA signaling, subsequently phosphorylated c-Raf on Ser259 site to block its activation, and then inhibited the extracellular regulated protein kinase (Erk)/mitogen-activated protein kinase (MAPK) signaling transduction, which finally prevented cell cycle progression and led to the suppression of VSMC proliferation [43]. The inhibition of VSMC proliferation might also be involved in SO_2_-mediated antihypertensive mechanisms.

### 4.2. SO_2_ and Pulmonary Hypertension

#### 4.2.1. SO_2_ and Hypoxic Pulmonary Hypertension

Pulmonary hypertension, characterized by high pressure in pulmonary artery, is a common complication of congenital heart disease (CHD), ultimately inducing right ventricular failure and even death. A prospective cohort study showed that the serum SO_2_ levels of children were, respectively, (10.6 ± 2.4), (8.9 ± 2.3), (7.3 ± 2.9), and (4.3 ± 2.1) *μ*M, in the control group, CHD without pulmonary hypertension group, CHD with mild pulmonary hypertension group, and CHD with moderate or severe pulmonary hypertension group [44], suggesting that a negative correlation existed between SO_2_ and pulmonary hypertension. Consistent with this, a downregulated SO_2_ level and AAT expression in lung tissue, accompanied with significant pulmonary hypertension, pulmonary vascular remodeling, and increased vascular inflammation, were found in rats under hypoxic condition [16, 45]. Most importantly, SO_2_ derivatives could markedly lower mean pulmonary artery pressure (mPAP) of hypoxic pulmonary hypertensive rats, whereas HDX advanced pulmonary hypertension [16, 45], indicating that decreased SO_2_/AAT pathway was involved in the development on hypoxic pulmonary hypertension. The hallmark pathological feature of hypoxic pulmonary hypertension is the pulmonary vascular structural remodeling including extracellular matrix accumulation, vascular smooth muscle proliferation, and inflammatory cells infiltrates [46]. SO_2_ derivatives prevented pulmonary vascular remodeling in hypoxic pulmonary hypertension through promoting collagen I and III degradation, suppressing abnormal collagen deposition in pulmonary vascular walls and through inhibiting pulmonary arterial SMC proliferation by downregulating Raf-1, MEK-1, and phosphorylating ERK under hypoxia [16]. Inflammation is important in the pathogenesis of hypoxic pulmonary hypertension. In addition, SO_2_ could inhibit pulmonary inflammation by suppressing expressions of nuclear factor-kappa B (NF-*κ*B) and intercellular adhesion molecule-1 (ICAM-1) [16], indicating the inhibitory effects of SO_2_ on inflammation may also be involved in the mechanism by which SO_2_ protects against hypoxic pulmonary hypertension.

#### 4.2.2. SO_2_ and Monocrotaline-Induced Pulmonary Hypertension

Monocrotaline (MCT), a pyrrolizidine alkaloid, increased mPAP and the ratio of right ventricle to left ventricle plus septum, coincident with the elevated SO_2_ content, AAT activity, and expression in rats [47]. SO_2_ derivatives injection significantly lowered mPAP and alleviated small and median pulmonary artery structural remodeling, whereas HDX which inhibited the activity of AAT and the production of endogenous SO_2_ further augmented mPAP, promoted right ventricular hypertrophy, and worsened pulmonary arteries structural remodeling [47]. These findings implied that the upregulation of endogenous SO_2_/AAT pathway might play a protective role in the development of MCT-induced pulmonary hypertension. The enhancement of oxidative stress is one of the main pathogenesis of MCT-induced pulmonary hypertension [48]. SO_2_ could upregulate the activities of antioxidative enzymes, including superoxide dismutase (SOD), glutathione peroxidase (GSH-Px), and catalase (CAT) in lung tissues and plasma from MCT-induced pulmonary hypertensive rats, whereas HDX decreased the activities of antioxidative enzymes [47]. These data suggested that the promotion of endogenous antioxidative capacities might be responsible for the protective role of SO_2_ in MCT-induced pulmonary hypertension.

#### 4.2.3. SO_2_ and High Pulmonary Blood Flow-Induced Pulmonary Hypertension

Severe pulmonary hypertension develops secondary to high pulmonary blood flow in patients with left-to-right shunt congenital heart defects or systemic arteriovenous shunt [49, 50]. However, the underlying mechanisms for flow-induced pulmonary hypertension remain poorly understood. The endogenous SO_2_/AAT2 pathway in pulmonary tissues was also inhibited in rats with pulmonary hypertension induced by high pulmonary blood flow [51]. SO_2_ reduced systolic pulmonary arterial pressure and improved pulmonary arterial structural remodeling, exhibiting decreased ratio of muscularized arteries to small pulmonary arteries and increased percentage of nonmuscularized arteries in the development of high pulmonary blood flow-induced pulmonary hypertension [51]. The mechanism was unclear, however. Both SO_2_ and H_2_S were derived from the methionine metabolism and they could convert to each other in mammals. Moreover, the endogenous H_2_S pathway exerted obvious mitigation effect on pulmonary hypertension induced by high pulmonary blood flow and H_2_S had strong vasodilating effect. Therefore, the researchers investigated the impact of SO_2_ on the endogenous H_2_S generating pathway in the pathogenesis of high blood flow-induced pulmonary hypertension. And they found that SO_2_ derivatives could upregulate the concentration of H_2_S in lung tissues, as well as the expressions of the key generating enzymes of H_2_S, including cystathionine *γ*-lyase (CSE), cystathionine *β*-synthase (CBS), and 3-mercaptopyruvate sulfurtransferase (3MST) [51]. Furthermore, SO_2_ increased the protein expression of these H_2_S producing enzymes probably through upregulating their gene transcription. These data suggested that SO_2_ alleviated pulmonary hypertension induced by high pulmonary blood flow in association with upregulating the reduced endogenous H_2_S pathway.

### 4.3. SO_2_ and Atherosclerosis

Atherosclerosis, a chronic and progressive pathological process in arteries, is a crucial pathological manifestation of cardiovascular diseases. Vascular inflammation, oxidative stress, VSMC proliferation, endothelial injury, and foam cell accumulation contribute to the formation of atherosclerotic plaque. Environmental toxicological study showed that the chronic exposure to gaseous air pollution such as SO_2_, NO, and CO might lead to the promotion of atherosclerosis [52, 53]. Growing evidence demonstrated that endogenous NO, CO, and H_2_S were beneficial in alleviating atherosclerosis [54–56]. They exerted significant anti-inflammation effect in the development of atherosclerosis, especially endothelium-derived NO which played a notably protective role in the early stage of atherosclerosis. However, the role of SO_2_ at physiological concentration in the development of atherosclerosis was unclear. Li et al. found that plasma and aortic SO_2_ contents were downregulated with the reduced aortic AAT activity in atherosclerosis rats [17], implying that the inhibition of SO_2_/AAT pathway might be involved in the pathogenesis of atherosclerosis. SO_2_ derivatives treatment diminished the size of atherosclerotic plaques in the coronary artery, not only by increasing H_2_S/CSE pathway and the NO/nitric oxide synthase (NOS) pathway, but also by elevating the antioxidative capacities through increasing plasma GSH-Px and SOD activities and decreasing MDA level [17]. Additionally, suppression of VSMC proliferation via cAMP/PKA signaling-mediated Erk/MAPK pathway might also contribute to the antiatherosclerotic effects of SO_2_ [43].

### 4.4. SO_2_ and Myocardial Ischemia Reperfusion

Myocardial ischemia-reperfusion (I/R) injury is an important cause of tissue and cell injury and often leads to heart failure. The main mechanisms involve inflammation, oxidative damage, and intracellular and mitochondrial calcium overload [57]. In rat myocardial I/R models made by ligating the left coronary artery for 30 min and reperfusion for 120 min, AAT1 protein expression was significantly decreased compared to sham operation group [18]. And SO_2_ derivatives preconditioning for 10 min before ischemia (with a low concentration of sulfur dioxide of 1–10 *μ*mol/kg) significantly decreased myocardial infract size and lowered levels of myocardial enzymes creatine kinase (CK) and lactate dehydrogenase (LDH) in plasma of rats with I/R injury* in vivo* [18]. SO_2_ preconditioning also increased cardiac function and attenuated myocardium apoptosis induced by I/R [18]. Ischemic preconditioning-induced endoplasmic reticulum stress (ERS) plays a protective role in the ischemia injury. Glucose-regulated protein 78 (GRP78), C/EBP homologous protein (CHOP), and phosphorylated eukaryotic initiation of the factor 2*α*-subunit (p-eIF2*α*) are the markers of myocardial ERS. SO_2_ pretreatment induced myocardial GRP78 expression and eIF2*α* phosphorylation prior to myocardial I/R, while inhibiting expressions of myocardial GRP78, CHOP, and p-eIF2*α* in rats with myocardial I/R [18]. Dithiothreitol, an ERS activator [58], mimicked the cardioprotective effect of SO_2_, whereas ERS inhibitor 4-phenylbutyrate abolished the cardioprotection of SO_2_ preconditioning [18, 59]. The above data suggested that augmentation of ERS by SO_2_ preconditioning before myocardial I/R contributed to cardioprotection against lethal ischemia. Moreover, SO_2_ preconditioning significantly elevated the phosphorylation of Akt and PI3K p85 and attenuated the myocardial damage in rats with I/R injury [60]. LY294002, a PI3K inhibitor, prevented the protective function of SO_2_ preconditioning as well as SO_2_-induced GRP78 and p-eIF2*α* expression [18, 60], indicating that PI3K/Akt signaling pathway likely mediated the activation of ERS by SO_2_ pretreatment in rats subjected to myocardial I/R. In addition, oxidative stress is involved in the pathogenesis of myocardial I/R. SO_2_ preconditioning with low dose of SO_2_ (1 and 5 *μ*mol/kg) prior to ischemia could significantly elevate plasma levels of SOD, GSH, and GSH-Px and reduce the MDA level [61], indicating that SO_2_ preconditioning enhanced the antioxidative capacity in rats with myocardial I/R. MAPK signaling, one of the most important pathways in cell signal transduction, is crucial to myocardial I/R. SO_2_ preconditioning significantly improved cardiac function and reduced myocardial expression of phosphorylated ERK1/2 protein in isolated rat heart with I/R [62]. Pretreated with PD98059, the ERK1/2 inhibitor abolished the above functions of SO_2_ [62]. These data indicated that inhibition of ERK1/2 signal pathway activation mediated the cardioprotection of SO_2_ preconditioning in isolated rat heart subjected to I/R. Taken together, elevation of PI3K/AKT signaling, suppression of ERK-MAPK pathway, augmentation of ERS, enhancement of antioxidative capacity, and attenuation of cardiomyocyte apoptosis might be involved in SO_2_-mediated cardiac protective mechanisms.

### 4.5. SO_2_ and Myocardial Injury

Myocardial injury is a common feature in various cardiac diseases. The underlying mechanisms include hypoxia, overactive oxidative stress, and calcium overload. A previous study found that endogenous SO_2_/AAT pathway was downregulated in isoproterenol- (ISO-) induced myocardial injury in rats [63]. Administration of SO_2_ (85 mg/(kg day)) could alleviate cardiac dysfunction and myocardial damage induced by ISO [63]. These data demonstrated that endogenous SO_2_ might be an important regulator in the pathophysiological process of myocardial injury. The molecular mechanisms underlying the cardioprotective effects of SO_2_ were still unknown. Oxidative stress was involved in the pathogenesis for ISO-induced myocardial injury. ISO produced oxygen free radicals caused membrane lipid peroxidation, injured the structure of cardiomyocytes, and finally resulted in myocardial damage [64]. But SO_2_ could increase myocardial antioxidant capacity in rats with myocardial injury by increasing the myocardial activity of SOD and GSH, upregulating the mRNA expression of SOD2 and GSH-Px1, and decreasing products of oxidative stress such as H_2_O_2_ and O_2_^∙−^ [63]. Oxidative stress could cause ERS in rat cardiomyocytes [65]. And the overactivated ERS would contribute to the development of myocardial injury. SO_2_ significantly inhibited the excessive activation of ERS, which might be involved in the mechanism by which SO_2_ derivatives protected against myocardial injury induced by ISO [66]. In addition, the products of oxidative stress cause the cardiomyocyte membrane damage and morphological mitochondrial injury. SO_2_ also attenuated ISO-induced mitochondrial swelling and deformation, which was important feature in apoptosis [63]. And cardiomyocyte apoptosis is a key pathological change in myocardial injury. Of note, supplementation of SO_2_ derivatives alleviated ISO-induced myocardial injury partly through reducing cardiomyocyte apoptosis [67]. The antiapoptotic function of SO_2_ was mediated by promoting bcl-2 expression, suppressing bax expression, enhancing mitochondrial membrane potential, inhibiting mitochondrion MPTP opening, reducing the release of cytochrome c from mitochondrion into cytoplasm, and decreasing the activation of caspase-9 and caspase-3 [67]. Therefore, the bcl-2/cytc/caspase-9/caspase-3 pathway was involved in the ISO-induced myocardial injury in rats. Intracellular calcium homeostasis exerts a fundamental effect on myocardial physiology and pathology. And calcium overload is an important mechanism involved in myocardial injury. SO_2_ treatment could inhibit the increased intracellular free Ca^2+^ concentration induced by ISO in H9C2 cells [68], indicating that the protective effect of SO_2_ in myocardial injury might be related to the calcium homeostasis regulated by SO_2_ in cardiomyocytes. Moreover, SO_2_ derivatives could modulate L-type calcium current and voltage-gated potassium channels in rat cardiomyocytes, indicating that ion channels might also be involved in the effect of SO_2_ on cardiomyocyte injury [69, 70].

## 5. Interaction among SO_**2**_ and Other Gasotransmitters

SO_2_ and H_2_S share the same endogenous substrate L-cysteine, and they can transform into each other under some biochemical condition [6, 23, 71]. Moreover, they share similar regulatory roles including vasorelaxation, antioxidative action, and inhibition of inflammation and apoptosis. Chen et al. found that SO_2_ upregulated the concentration and production of H_2_S in hypoxic rats. And SO_2_ increased the expression of CSE and 3MST in pulmonary arteries of hypoxic pulmonary hypertensive rats [72]. In addition, SO_2_ alleviated pulmonary hypertension and improved the pulmonary vascular pathological injury induced by high pulmonary blood flow in association with upregulating the endogenous H_2_S pathway [51]. Furthermore, SO_2_ derivatives have a marked antiatherogenic effect with an increased aortic H_2_S/CSE pathway in atherosclerotic rats [17]. In rats with myocardial I/R injury, SO_2_ preconditioning markedly upregulated the myocardial H_2_S level and CSE expression [61]. The above findings provide some evidence that there is a crosstalk between SO_2_ and H_2_S. Moreover, NO also shares a variety of the similar biological effects of H_2_S and SO_2_, including vasodilation, antioxidation, and anti-inflammatory actions. Li and Meng found that a low concentration (3 or 5 nM) of a NO donor sodium-nitroprusside enhanced the vasodilating effect of SO_2_ by nearly sixfold [26], suggesting that SO_2_ and NO have a synergistic effect on vasodilation. In contrast, the NOS inhibitor L-NAME could abolish the vasorelaxing effect of SO_2_ derivatives (0.5 and 1 mM) in endothelium-intact rings, indicating that endothelium-dependent vasorelaxation induced by SO_2_ was partially mediated by a NOS pathway [73]. Additionally, both acute and prolonged SO_2_ exposure upregulated the eNOS-NO-cGMP pathway, which might be involved in the vasodilation induced by SO_2_ [34]. Moreover, SO_2_ increased vasorelaxation in SHRs by enhancing the vasorelaxing response to NO and upregulating NO production in aortic tissues [40]. And SO_2_ also increased NO/NOS pathway in rats with atherosclerosis [17]. By contrast, SO_2_ pretreatment reduced the myocardial tissue levels of NO and expression of iNOS in rats with I/R [61]. These data suggest that there is also an interaction between SO_2_ and NO. Hence, endogenous SO_2_ participates in crosstalk with H_2_S and NO and an endogenous gaseous messenger molecule network might exist in mammals. However, there are still many questions to be answered about the interactions among these gases. For example, the exact pertinence among these gases in the various pathways of cardiovascular protection has not been fully explored. It is also not known if a combination of these gases will provide synergistic effects in the therapy of cardiovascular diseases. Therefore, additional studies are needed to further investigate interactions among the gasotransmitter pathways.

## 6. Conclusions

In summary, SO_2_ can be generated in the cardiovascular system of mammals and the SO_2_/AAT pathway participates in many biological functions [22, 74]. Endogenously derived SO_2_ or SO_2_ derivatives at physiological concentrations play a crucial role in normal physiological process including regulation of vascular tone and cardiac function. In addition, SO_2_/AAT pathway has important pathophysiological significance in many cardiovascular diseases, such as hypertension, pulmonary hypertension, atherosclerosis, ischemia-reperfusion injury, and myocardial injury. Just as NO, carbon monoxide (CO), and H_2_S, SO_2_ is also an endogenous gaseous signaling molecule in the cardiovascular system [71, 75]. However, the biological mechanisms by which endogenous SO_2_ regulates different cardiovascular diseases and the further cardiovascular effects of SO_2_ still need to be deeply investigated.

Clarifying the interactions among SO_2_ and other endogenous gasotransmitters could improve clinical translation. SO_2_ could upregulate endogenous level of H_2_S or NO in several cardiovascular diseases such as atherosclerosis, systemic hypertension, or pulmonary hypertension [17, 40, 51]. These lines of evidence imply a crosstalk among SO_2_ and other gasotransmitters (NO, CO, and H_2_S) in the cardiovascular system, which requires further exploration.

An understanding of the cardiovascular protective function of SO_2_ may lead to a new therapeutic strategy based on the modulation of SO_2_ production. Thus, the function and signaling pathway relating to AAT in the cardiovascular system are worthy of further investigation. Additionally, the design of SO_2_-controlled releasing agents under physiological condition is extremely urgent, because the stable and reliable SO_2_ donors are not only the useful research tools, but also potential therapeutic agents to treat cardiovascular diseases. Nowadays, the majority of cardiovascular studies on SO_2_ have been performed in rats and mice, which lack clinical evidence. Exploring the role of SO_2_ in large animal models with similar cardiovascular features as human suffering from cardiovascular diseases would help a transition to clinical trials.

## Abbreviations

SO_2_: Sulfur dioxide
AAT: Aspartate aminotransferase
I/R: Ischemia-reperfusion
CDO: Cysteine dioxygenase
H_2_S: Hydrogen sulfide
NO: Nitric oxide
CO: Carbon monoxide
HDX: L-Aspartate-*β*-hydroxamate
L-Ca^2+^: L-type calcium
BK_Ca_: Big-conductance Ca^2+^-activated K^+^
K_ATP_: Adenosine triphosphate-sensitive potassium
cAMP: 3′-5′-Cyclic adenosine monophosphate
PGI_2_: Prostacyclin
AC: Adenylyl cyclase
PKA: Protein kinase A
cGMP: 3′-5′-Cyclic guanosine monophosphate
LVDP: Left ventricular developed pressure
PKC: Protein kinase C
SHR: Spontaneously hypertensive rats
eNOS: Nitric oxide synthase
VSMCs: Vascular smooth muscle cells
PDGF-BB: Platelet-derived growth factor-BB
Erk 1/2: Extracellular regulated protein kinase 1/2
MAPK: Mitogen-activated protein kinase
CHD: Congenital heart defects
mPAP: Mean pulmonary artery pressure
NF-*κ*B: Nuclear factor-kappa B
ICAM-1: Intercellular adhesion molecule-1
MCT: Monocrotaline
SOD: Superoxide dismutase
GSH-Px: Glutathione peroxidase
CAT: Catalase
CSE: Cystathionine *γ*-lyase
CBS: Cystathionine *β*-synthase
3MST: 3-Mercaptopyruvate sulfurtransferase
NOS: Nitric oxide synthase
CK: Creatine kinase
LDH: Lactate dehydrogenase
GRP78: Glucose-regulated protein 78
CHOP: C/EBP homologous protein
eIF2*α*: Eukaryotic initiation of the factor 2*α*-subunit
p-eIF2*α*: Phosphorylated eIF2*α*
ERS: Endoplasmic reticulum stress
ISO: Isoproterenol.
